# Factors associated with BMI, underweight, overweight, and obesity among adults in a population of rural south India: a cross-sectional study

**DOI:** 10.1186/s40608-016-0091-7

**Published:** 2016-02-20

**Authors:** Matthew Little, Sally Humphries, Kirit Patel, Cate Dewey

**Affiliations:** Department of Population Medicine, University of Guelph, Guelph, ON Canada; Department of Sociology and Anthropology, University of Guelph, Guelph, ON Canada; Department of International Development Studies, Menno Simons College, University of Winnipeg, Winnipeg, MB Canada

**Keywords:** Underweight, Overweight, Obesity, Physical activity, Diet, Rural, India, Tamil Nadu, Diabetes

## Abstract

**Background:**

Overweight, obesity, and related chronic diseases are becoming serious public health concerns in rural areas of India. Compounded with the existing issue of underweight, such concerns expose the double burden of disease and may put stress on rural healthcare. The purpose of this article was to present the prevalence and factors associated with underweight, overweight, and obesity in an area of rural south India.

**Methods:**

During 2013 and 2014, a random sample of adults aged 20–80 years were selected for participation in a cross-sectional study that collected information on diet (using a food frequency questionnaire), physical activity (using the Global Physical Activity Questionnaire), socioeconomic position (using a wealth index), rurality (using the MSU rurality index), education, and a variety of descriptive factors. BMI was measured using standard techniques. Using a multivariate linear regression analysis and multivariate logistic regression analyses, we examined associations between BMI, overweight, obesity, and underweight, and all potential risk factors included in the survey.

**Results:**

Age and sex-adjusted prevalence of overweight, obesity class I, and obesity class II were 14.9, 16.1, and 3.3 % respectively. Prevalence of underweight was 22.7 %. The following variables were associated with higher BMI and/or increased odds of overweight, obesity class I, and/or obesity class II: Low physical activity, high wealth index, no livestock, low animal fat consumption, high n-6 polyunsaturated fat consumption, television ownership, time spent watching television, low rurality index, and high caste. The following variables were associated with increased odds of underweight: low wealth index, high rurality index, and low intake of n-6 PUFAs.

**Conclusion:**

Underweight, overweight, and obesity are prevalent in rural regions of southern India, indicating a village-level dual burden. A variety of variables are associated with these conditions, including physical activity, socioeconomic position, rurality, television use, and diet. To address the both underweight and obesity, policymakers must simultaneously focus on encouraging positive behaviour through education and addressing society-level risk factors that inhibit individuals from achieving optimal health.

## Background

Obesity and its associated health effects are quickly becoming serious health concerns in low- and middle-income countries [1]. In 2010, the World Obesity Federation estimated that over one billion adults were overweight and 475 million were obese globally [2], the majority of which resided in developing countries in Latin America, Africa, and Asia. In India, successive studies suggest obesity is increasing rapidly, with recent prevalence estimates upwards of 15 % [3]. The sedentary and dietary effects of urbanization and modernization are often blamed for propagating overweight and obesity [4], however little research has examined the risk factors contributing to rising prevalence in rural areas where 70 % of the Indian population live and modernization has occurred less rapidly [5]. In-depth examinations of overweight and obesity in rural regions are therefore essential, especially considering these regions are often compromised by low literacy and poor access to healthcare services [6].

Overweight and obesity are associated with increased risk of non-communicable diseases such as metabolic syndrome, high cholesterol, type 2 diabetes mellitus, high blood pressure, and cardiovascular disease; conditions that are already serious public health concerns in rural and urban India alike [5, 7]. Studies of non-communicable disease biomarkers have long conferred the possibility of an ‘Asian Indian phenotype’ that produces higher-risk central adiposity at a lower body-mass index (BMI) than comparable populations in Europe and North America. For example, Razak et al. [8] found that average fasting glucose levels, LDL cholesterol concentrations, and blood pressure found in Europeans at a BMI of 30 kg/m^2^ could be found in Asian Indians at a much lower BMI. Such studies indicate that Asian Indians are more susceptible to the negative health consequences of overweight and obesity. Even more troubling is the co-occurrence of undernutrition and overnutrition in rural villages and households, termed the ‘double burden’ of disease, which serves to exacerbate poverty and limit economic growth in these areas [9]. While a number of studies have examined associations between dietary and lifestyle factors and prevalence of underweight, overweight, and obesity in the US [10, 11] and EU [12, 13], little research has examined similar associations in India, and even fewer in rural India [5].

The present study was conducted from November 2013 to March 2014 and examined a random sample of 753 people living in a rural region of northern Tamil Nadu. The objectives of the study were: (1) to determine the prevalence of underweight, overweight, and obesity among the study population; and (2) to determine the risk factors that affect BMI, underweight, overweight, obesity in rural regions of India using forwards stepwise linear and logistic regression models. The study was carried out in collaboration with the Development for Humane Action Network (DHAN) Foundation as part of a DFATD/IDRC-funded project entitled “Revalorizing small millets: Enhancing the food and nutritional security of women and children in rainfed regions of South Asia using underutilized species”.

## Methods

### Ethics, consent, and permissions

We obtained clearance for the study from the University of Guelph Research Ethics Board (certificate reference number 12MY019). Permission for the study was granted by the High Commission of India in Ottawa, Canada. Upon arrival to the research site, and prior to the recruitment process, we approached local authorities (*panchayat* councils, local police officials, and hospital medical staff) and sought and obtained permission to carry out the study. Informed consent to participate in the study was obtained from all participants prior to their enrollment.

### Sampling frame and recruitment

The sampling frame consisted of the entire adult population (>19 years old) of two rural *panchayat* wards (Anchetty panchayat and Madakkal panchayat), in the Krishnagiri District of Tamil Nadu. The region is comprised of several small villages surrounding the central market village of Anchetty.

Our target was to sample 800 participants following a sample size calculation for a sub-study published elsewhere [14]. A randomized two-stage recruitment method was employed, in which we first approached a random sample of 8 % of households in the sampling frame, then employed the WHO’s Kish method to select a single household member for the study [15, 16]. If the selected individual refused, we removed them from the list of occupants and employed the Kish method again until the selected individual agreed to participate. If all adult members of the household were not present or refused, we moved to an immediately neighbouring household to recruit the required sample. All absences and refusals were considered non-responses in calculating response rate. We recorded the reason for non-response whenever possible. After securing oral consent to participate in the study, we organized a follow-up for completion of surveys and collection of health outcome data. One of either a doctor or a nurse collected anthropometric measurements and one of three nutritionists (one male, two female) conducted all interviews. Health care practitioners and nutritionists were gender-matched with participants to reduce potential response bias.

### Anthropometric measurements and descriptive questionnaire

Standing height was measured at end of expiration against a flat wall using a ruler pressed against the crown of the head and a measuring tape. Weight was measured in light clothes with bare feet using a household digital scale (NOVA™ BGS1207 model). Blood pressure measurements were recorded as the average of two readings using an Omron™ BP786-10 handheld electronic blood pressure monitor in the sitting position using the right upper arm and one of three sized cuffs after a period of 5 min sitting. Participants completed a structured questionnaire about age, sex, occupation, education, medical history, tobacco use, socioeconomic status, physical activity, and dietary intake.

### Socioeconomic status wealth index

We created a wealth index using a modified subset of 13 of 29 questions taken from the Standard of Living Index used by the 2nd round of the National Health and Family Survey [17]. We selected those questions we believed to be most relevant for our study population. They comprised both household and village characteristics, including: electrification (electricity, kerosene, gas or oil, other source of lighting), source of drinking water (pipe, hand pump, well in residence, public tap, hand pump, public well, or other water source), type of toilet facility (own flush toilet, public or shared flush toilet or own pit toilet, shared or public pit toilet, no facility), type of house (pucca, semi-pucca, or kutcha), cooking fuel (electricity, natural gas, kerosene, or wood). In addition, we collected information on ownership of house, land, livestock (cattle, chickens, goats, sheep, and buffalo), ceiling fan, radio, vehicle (bicycle, moped, motorcycle, or car), and television. Each attribute was weighted to give a maximum score of 36. Weights of items were developed by the International Institute of Population Sciences in India based on a priori knowledge about the relative significance of the items in determining SES [18]. Due to the rural subsistence nature of the local economy, this asset-based score is considered a more appropriate indicator of SES than education, income, or occupation [17]. For the remainder of the article, we will refer to this score as the ‘wealth index’.

### Rurality index

All previous epidemiology studies in India known to the authors define households as either rural or urban based on a dichotomous typology, which fails to adequately capture the variability inherent in the urban/rural continuum [19]. We created a rurality index to quantify the degree of rurality of each household. This index was based on that developed for the USA by Weinert and Boik (1995) and adapted for use in India [20]. The index was based on two factors: (1) distance to primary health centre, and (2) population of village in which household resides. We collected and standardized these values, then combined them, assigning a half weighted positive value to ‘distance to healthcare facility’ and a full weighted negative value to ‘household population village’.

### Physical activity assessment

We conducted the Global Physical Activity Questionnaire (GPAQ) with each participant, designed by the World Health Organization (WHO) as part of the WHO STEPwise approach to chronic disease risk factor surveillance [21]. GPAQ comprises 19 questions grouped to assess individuals’ physical activity behaviour in three main domains over the course of 1 year: work, travel, and recreation. The GPAQ was paired with locally relevant photographs depicting ‘moderate’ and ‘vigorous’-intensity work and recreation activities. In addition, we collected time spent sitting and time spent watching television per day. Physical activity scores were calculated using WHO’s GPAQ Analysis Guide [21], which provided a total measure of Metabolic Equivalent (MET) minutes per week based on amount of time spent performing moderate (assigned 4 MET equivalents per minute) and vigorous physical activity (assigned 8 MET equivalents per minute) for work, transport, and recreation purposes. We scaled values to hours per day of moderate physical activity for easier interpretation.

### Dietary and nutrition assessment

Diet was assessed using a south Indian food frequency questionnaire (FFQ), validated for Tamil Nadu by the Madras Diabetes Research Foundation (MDRF) [22]. MDRF provided permission to use the research tool and organized a 2-day training program for our three nutritionists on how to properly administer the FFQ, record responses, and enter data using their data entry software model. The FFQ uses a food atlas with photographs of dishes and serving sizes to collect information about the frequency of consumption of 212 dishes and foods. This tool is paired with EpiNu® [23], a software program that calculates overall intake of food categories and macro- and micronutrients based on frequency of consumption of dishes and laboratory-based nutritional analyses for each dish [24]. After completion of the study, FFQ data was analyzed by MDRF using EpiNu®, providing us with detailed data about dietary and nutrient intake for each participant. Energy intake was analyzed as kilocalories (kcal) consumed per day. All other dietary intake variables were scaled to grams per 1000 kcal consumed.

### Definitions

Literacy was defined as self-professed fluency in reading and writing. Tobacco consumers were defined as individuals who currently smoked at least one cigarette and/or chewed *paan* at least once per day. High blood pressure was defined as mean systolic blood pressure ≥ 140 mm Hg and/or mean diastolic blood pressure ≥ 90 mm Hg and/or treatment with blood pressure medication [25]. For each participant we calculated the body mass index (BMI) as the weight in kilograms divided by the squared height in meters (kg/m^2^).

As per the definitions of the International Obesity Task Force (IOTF), underweight was defined as BMI < 18.5 kg/m^2^, overweight was defined as BMI ≥ 23.0 kg/m^2^ but <25 kg/m^2^, obesity class I was defined as BMI ≥ 25 kg/m^2^ and < 30 kg/m^2^, and obesity class II was defined as BMI ≥ 30 kg/m^2^ [26]. Low caste was analyzed as a binary variable with Scheduled Castes (SC) and Scheduled Tribes (ST) being low caste and all others as an amalgamated referent. High caste was analyzed as a binary variable, with Brahmins being high caste and all other castes as an amalgamated referent.

### Statistical analysis

We identified outliers, cleaned the data set, and completed summary statistics in Microsoft Excel 10.0 [27]. No values were considered outliers after correcting data entry errors. As such, all observations were included in the analysis.

Underweight, overweight, and obesity prevalence data were standardized using state-level age and sex data from the 2011 national census [28]. In separate models, univariate associations between sex and BMI category were analyzed. Since there were no significant associations between sex and outcome variables, we present the results for men and women combined. Means of descriptive characteristics, socioeconomic and education characteristics, physical activity habits, and dietary intake were calculated across categories of BMI, including underweight, normal, overweight, obesity class I, and obesity class II. Values were expressed as mean +/- SD or percentages. One-way analysis of variance (for continuous variables) and Pearson’s *X*^2^ test were used to examine differences across outcome groups. Following this, three multivariate regression models were built in STATA 13.0 [29].

#### Model one

We employed a forward stepwise linear regression model-building process to determine associations between BMI as a continuous variable and putative risk factors. All factors were first analyzed in univariate regressions (see Appendix 1 for full list of factors). Variables with significant associations (*p*-value < 0.2) in univariate analyses were considered for an initial multivariate linear regression model. We then employed a forward stepwise model building process and eliminated non-significant variables (using a *p*-value cut-off of 0.05) from the multivariate model, assuming a lack of confounding if coefficients of all remaining variables did not change by more than 20 % after addition or removal of the potential confounder [30]. Quadratic terms and interaction terms were assessed if there were biological or practical reasons to believe they may be significant. BMI was log-transformed to improve normality of residuals and homoscedasticity. We ensured the transformed model obeyed the assumptions of linear regression models, including independence of variables, normality of residuals (using a Shapiro-Wilks test and quartile quartile plots), and homoscedasticity (using a Cook-Weisberg test and a residual-fitted value plot).

#### Model two

In a second step, we fit a multinomial logistic regression with overweight, class I obesity, and class II obesity as separate outcome groups. Underweight and normal weight classes were combined into a referent category to increase the statistical power of the analysis [31]. We examined the same variables as those assessed in the linear regression analysis (Appendix 1). Variables were first assessed for significance in multinomial univariate logistic analyses. All variables with associations (*p*-value <0.2) with any of the outcome groups were included in the initial multivariate analysis. We then employed a forward stepwise process and eliminated non-significant variables (*p*-value <0.05) using the same methods as those used in model one to determine adjusted odds ratios (ORs) and 95 % confidence intervals (CIs).

#### Model three

In a final step, we fit a multivariate logistic regression with underweight as an outcome group. All other weight categories were combined as the referent group [32]. We examined the same variables as those assessed in the models one and two (see Appendix 1). We then followed the same methods as used in model two in order to determine adjusted ORs and corresponding CIs for each significant variable (*p*-value less than 0.05) in the final model.

## Results

Of the 812 individuals recruited for the study, 753 (92.7 %) participated (341 men and 412 women), of whom 752 (92.6 %) completed an FFQ and 745 (91.7 %) consented to anthropometric measurements. Response rate was 87.4 % among men and 99.2 % among women. Disparity in the response rate was primarily due to migration among local men and thus unavailability at the time of sampling. The mean age was 47 ± 14.7 and the literacy rate was 35.1 %. Crude prevalence rates of underweight, overweight, obesity class I, and obesity class II were 22.7, 15.8, 17.7, and 3.5 %, respectively. Age- and sex-adjusted prevalence of underweight, overweight, obesity class I, and obesity class II were 22.7, 14.9, 16.1, and 3.3 % respectively.

Baseline characteristics for the study population by BMI category are outlined in Table 1. A significant difference was seen across categories in several characteristics, including socioeconomic status, education, physical activity, and sitting time. Intake of carbohydrates, total fat, dietary fibre, pulses, and fruits and vegetables was significantly different by BMI category.

**Table 1:** Baseline characteristics of a sample of individuals in rural south India by BMI category

Characteristic	Underweight (BMI < 18.5 kg/m^2^, *n* = 84)	Normal (*n* = 300)	Overweight (BMI ≥ 23.0 kg/m^2^ and <25 kg/m^2,^ *n* = 118)	Obesity class I (BMI ≥ 25 kg/m^2^ and < 30 kg/m^2^, *n* = 132)	Obesity class II (BMI ≥ 30 kg/m^2^, *n* = 26)	*p*-value for trend
*Descriptive characteristics*						
Age	49.6 ± 17.2	46.8 ± 14.8	47.4 ± 13.5	45.1 ± 11.7	43.6 ± 11.5	0.05
Women (%)	51.7	53.8	52.5	58.3	76.9	0.145
Waist to hip ratio	0.84 ± 0.072	0.87 ± 0.075	0.91 ± 0.07	0.92 ± 0.09	0.92 ± 0.08	<0.001
Hypertension (%)	19.4 %	25.8	40.7	38.9	50	<0.001
Rurality Index	0.04 ± 1.1	−0.37 ± 1.3	−0.86 ± 1.32	−1.0 ± 1.28	−1.95 ± 1.0	<0.001
Current tobacco consumer (%)	50	40.5	34.7	31.5	7.7	<0.001
*Socioeconomic and education characteristics*						
Wealth index	9.7 ± 3.9	10.7 ± 4.6	11.3 ± 4.8	12.3 ± 5.0	13.5 ± 5.6	<0.001
Vehicle ownership (%)	17.6	26.3	34.7	35.6	42.3	0.001
Television ownership (%)	78.3	88.3	95.8	95.5	96.1	<0.001
Pucca housing (%)	4.5	12.2	15.3	22.7	47.1	<0.001
In-house tap water (%)	4.5	7.6	6.7	10.6	30.8	<0.001
Education (grade achieved)	2.1 ± 3.5	3.1 ± 4.4	3.2 ± 4.2	3.7 ± 4.5	4.4 ± 4.8	0.007
*Physical activity characteristics*						
Physical activity (hours/day of moderate physical activity)	4.0 ± 3.4	4.4 ± 3.7	4.3 ± 4.0	3.2 ± 3.4	1.1 ± 2.3	<0.001
Sitting time (hours/day)	4.2 ± 2.6	4.3 ± 4.3	4.3 ± 2.5	5.0 ± 2.8	6.2 ± 2.8	<0.001
Television time (hours/day)	1.3 ± 1.35	1.3 ± 1.2	1.6 ± 1.2	1.8 ± 1.37	2.1 ± 1.6	<0.001
Labour occupation (%)	63.6	64.4	56.7	51.6	38.5	0.012
*Dietary characteristics*						
Current alcohol consumer (%)	49.4	46.2	52.5	44.7	34.6	0.432
Total energy intake (kcal/day)	2390 ± 758	2365 ± 724	2439 ± 625	2440 ± 702	2031 ± 585	0.11
Carbohydrates (g/1000 kcal)	183 ± 14	181 ± 15	177 ± 15	177 ± 13	170 ± 8	<0.001
Protein (g/1000 kcal)	25.5 ± 1.9	25.7 ± 2.0	25.5 ± 1.8	25.9 ± 1.7	26.2 ± 1.8	0.26
Total fat (g/1000 kcal)	18.2 ± 5.3	19.2 ± 5.3	20.6 ± 4.8	21.2 ± 5.1	23.6 ± 2.9	<0.001
Dietary fibre (g/1000 kcal)	34.4 ± 12	32.7 ± 13	29.3 ± 11	29.6 ± 11	23.1 ± 7.7	<0.001
Dairy products (g/1000 kcal)	85.2 ± 77	74.1 ± 64	79.7 ± 62	87.1 ± 67	102.5 ± 66	0.11
Pulses and legumes (g/1000 kcal)	24.2 ± 11	27.6 ± 12	29.6 ± 12	29.4 ± 12	30.7 ± 11	0.01
Meat and poultry (g/100 kcal)	2.93 ± 3.8	3.3 ± 4.1	2.8 ± 3	3.3 ± 4.6	3.7 ± 3.4	0.61
Fruits and vegetables (g/1000 kcal)	63.1 ± 41	75.6 ± 46	86.6 ± 58	84.3 ± 50	87.0 ± 37	<0.001
Refined grains (g/1000 kcal)	135 ± 76	153 ± 83	178 ± 90	165 ± 75	154.6 ± 55	<0.001

### Model one: linear regression of BMI

The multivariate linear regression model (Table 2) had an R-squared value of 0.20, indicating 20 % of the variance in BMI among participants was explained by the variables included in the model. After adjustment for all potential confounders, there existed positive associations between BMI and wealth index, TV time, n-6 polyunsaturated fatty acid (PUFA) consumption, and high caste. There were inverse associations between BMI and physical activity, livestock ownership, rurality, animal fats consumption, and tobacco consumption. We repeated the analysis using log-transformed BMI to improve normality of residuals and homoscedasticity. As the results were very similar, we present here the fitted coefficients without transformation for ease of interpretation [33].

**Table 2:** Factors associated with body mass index for a sample of individuals living in rural south India based on a multivariable analysis

Variable	Coefficient	Standard error	*P* value
Constant	19.48	0.57	<0.001
Physical activity (h/day of moderate activity)	−0.085	0.040	0.034
Wealth index	0.14	0.031	<0.001
Livestock ownership (Y/N)	−0.76	0.30	0.013
Animal fats consumption (g/1000 kcal)	−0.060	0.026	0.023
n-6 PUFA consumption (g/1000 kcal)	1.36	0.59	0.022
Television time (h/day)	0.34	0.11	0.003
Tobacco consumption (Y/N)	−0.95	0.29	0.001
MSU rurality index	−0.76	0.12	<0.001
High caste (Y/N)	1.64	0.49	0.001

### Model two: multinomial logistic regression of overweight and obesity

Table 3 shows factors associated with overweight, obesity class I, and obesity class II based on a multinomial logistic regression. Normal and underweight individuals were amalgamated into the referent group. The model was not adjusted for age and gender because neither was significantly associated with any outcome group in the final model.

**Table 3:** Factors associated with overweight, class I and class II obese for a sample of individuals in rural south India

Risk factor	Overweight (23–25 kg/m^2^) OR 95 % CI	Obesity class I (25–30 kg/m^2^) OR 95 % CI	Obesity class II (>30 kg/m^2^) OR 95 % CI
Physical activity (h/day of moderate activity)	1.02 (0.96, 1.08)	0.94^b^ (0.88, 0.99)	0.75^a^ (0.62, 0.92)
Wealth index	1.02 (0.97, 1.07)	1.07^a^ (1.03, 1.12)	1.09^b^ (1.00, 1.17)
Own a television (Y/N)	2.88^b^ (1.08, 7.67)	2.19 (0.81, 5.87)	1.17 (0.13, 10.8)
Television time (h/day)	1.06 (0.90,1.26)	1.19^b^ (1.01, 1.39)	1.22^c^ (0.13, 10.8)
High caste (Y/N)	2.37^b^ (1.21, 4.67)	3.54^a^ (1.90, 6.60)	4.62^b^ (1.22, 17.49)
Rurality index	0.70^a^ (0.52, 0.83)	0.69^a^ (0.59, 0.82)	0.41^a^ (0.26, 0.63)
Tobacco consumption	0.80 (0.52, 1.23)	0.75^c^ (0.48, 1.16)	0.15^b^ (0.035, 0.68)

^a^significant to *p* < 0.01

^b^significant to *p* < 0.05

^c^tendency to *p* < 0.2

### Model three: multinomial logistic regression of underweight

Table 4 shows the factors associated with underweight determined by a logistic regression. Only three variables were significantly associated with odds of underweight in the final model.

**Table 4:** Factors associated with underweight for a sample of individuals in rural south India

Risk factor	Odds Ratio (95 % CI)
Wealth index	0.93^a^ (0.90, 0.98)
Rurality index	1.48^a^ (1.24, 1.56)
n-6 PUFA consumption (g/1000 kcal)	0.98^b^ (0.95, 0.99)
Tobacco consumption	0.77^a^ (0.43, 1.38)

^a^significant to *p* < 0.01

^b^significant to *p* < 0.05

## Discussion

Age and sex-adjusted prevalence of underweight, overweight, obesity class I, and obesity class II were 22.7, 14.9, 16.1, and 3.3 %, respectively. Prevalence rates of underweight, overweight, and obesity class I were not significantly different between women and men. Obesity class II was more common among women (*p* < 0.05), a finding that is consistent with several other studies in India [34, 35]. Age and sex-adjusted prevalence of underweight was lower than the latest (2005) national rural estimate of 35 %, indicating less undernutrition in the study site than elsewhere in India [3]. Age and sex-standardized prevalence of obesity was higher than the latest national rural estimate of 6 % [34], which is likely partially due to different BMI cut-off values and age differences in the sample population. Prevalence rates of overweight and obesity were comparable to more recent studies in rural south India that used similar cut-off values and age categories [34, 36]. For example, Misra et al. (2011) found prevalence of overweight and obese were 14 and 18 % in rural Tamil Nadu [35].

The simultaneous high prevalence of underweight and overweight/obesity indicates continued existence of the ‘double burden of disease’ as described by Doak et al. (2005) [8]. While we are unable, due to sampling methods, to determine if underweight and overweight persons cohabit the same households, our findings do indicate the coexistence of underweight and overweight at the village level. Similar results have been found in agricultural regions of Brazil [37], South Africa [38], Russia [39], and China [40]. With age and sex-standardized prevalence rates of underweight and overweight/obesity accounting for over half the study population, and the potential for future increases in prevalence of overweight/obese as well as complications due to associated non-communicable diseases, this double burden should be considered a major concern that threatens to strain the limited health care services in the region.

### Correlates of underweight, overweight, and obesity

While most studies examining risk factors contributing to underweight, overweight, or obesity categorize or dichotomize BMI and analyze associations using logistic regressions (e.g. [6, 12, 13, 41]), there is a large body of literature suggesting that categorizing continuous variables isn’t statistically effective as it results in a loss of information and power due to a reduction of variability in each category [42–44]. For this reason, we conducted linear regressions in addition to logistic regressions. We found strong independent associations between several risk factors and BMI, underweight, overweight, and obesity.

#### Physical activity

In the unadjusted univariate model, each hour of moderate physical activity was associated with a 0.095 kg/m^2^ decrease in BMI. After adjusting for other variables, each hour of moderate physical activity was associated with a 0.085 kg/m^2^ decrease in BMI. This is equivalent to -231 g for a typical Indian man (165 cm tall) and -196 g for a typical Indian woman (152 cm tall) (based on height data from Mamidi et al. 2011 [45]). Although the magnitude of this difference may seem small, when the coefficient was applied to the values for low (10th percentile of PA scores) versus high (90th percentile), the model predicted a difference of -2.34 kg for a typical man and -1.99 kg for a typical woman. In addition, higher physical activity was associated with lesser odds of obesity class I and obesity class II. These findings support the ideas raised by Shetty et al. (2002) that reduced physical activity is one of the main causes of overweight and obesity in India [46]. Due to the cross-sectional nature of the study, we are unable to determine if reduced physical activity is a cause or a consequence of weight gain [27, 47]. Physical activity was not associated with underweight despite suggestions by Hausenblas and Downs that excessive exercise (e.g. forced labour) could lead to underweight and wasting [48, 49]. However, it is unlikely that such individuals were captured in the study.

Several researchers suggest that modernization has contributed to declining physical activity in a number of ways. An influx of vehicles has reduced the need for active transportation [5]. Mechanization of farming processes has resulted in less need for strenuous labour. For example, Ramachandran et al. (2004) found a 57.2 % decrease in the number of individuals engaged in manual labour in a rural population of eastern Tamil Nadu between 1989 and 2003 [50]. Exercise for leisure is not common in rural India, so reduced physical activity for work or travel is rarely offset by recreational activities [51]. Taking into account the growing prevalence of overweight and obesity in India, our findings are consistent with the view that physical activity may reduce or prevent increases in body weight. Our results suggest that even a modest level of activity may confer health benefits.

#### Rurality index

The Census Bureau of India classifies households as either urban or rural and policymakers enforce these definitions in health, food, and economic policies [52]. Researchers in India have adopted this definition when examining the effects of urbanization on chronic disease outcomes, often making comparisons between rural and urban populations [35, 53–56]. However, a dichotomous definition of rurality is ineffective when examining rural or urban populations separately. In addition, it fails to account for the gradient of rurality, homogenizes a heterogeneous population, and oversimplifies the effects of rurality as a descriptive factor [57]. Indeed, as stated by Rousseau (2005), ‘rural’ should not imply a single community but a wide range of communities with various features [58]. A standardized definition of rurality has thus proved elusive, as the essence of ‘rural’ villages is a complex and context-specific interplay of culture, affluence, geography, agriculture, and access to markets. Several studies have confirmed that urban status is associated with higher BMI and greater odds of overweight and obesity and rural status is associated with greater odds of underweight [35, 53–55]. Upon deeper exploration, many of these studies described associations between urban or rural status and other more proximate risk factors, such as income, physical activity, and diet (e.g. Pandey et al. 2013 [54]). While in our study, the entire study region is classified as rural, we were interested in the more nuanced conceptualization of a rural–urban continuum [59, 60]. In light of the many public health problems posed by urbanization, we believe it prudent to quantify and examine this concept in an epidemiologic capacity.

Researchers in the United States [20], Canada [61, 62], Australia [63], and Britain [64] have developed localized rurality indices intended to quantify the rural–urban continuum for researchers, policymakers, and healthcare providers. However, no rurality index has been developed for India. We elected to adapt the Montana State University (MSU) rurality index [20] due to its simplicity and relevance to the Indian context. Our results showed greater rurality was associated with lower BMI and decreased odds of overweight, class I obesity, and class II obesity. In addition, increased rurality was associated with greater odds of underweight. We therefore posit that degree of rurality is accompanied by a gradient of lifestyle and dietary differences not captured by other measures. In addition, individuals living in larger villages and closer to the market may experience processes associated with urbanization while still maintaining their rural status as per the Indian definition. Some studies have suggested urban residents face greater stress loads and higher prevalence of depression, which in turn affects physical health and possibly BMI [65, 66]. As we did not collect or analyze any measure of stress, perhaps the effects of such ‘hidden’ risk factors are captured by the rurality index. Further research on rurality and health outcomes is crucial to encourage policymakers to reevaluate the simplistic rural/urban dichotomy upon which most health, food, and economic policy is founded.

#### Socioeconomic status (SES)

The asset-based wealth index, analyzed as a proxy for SES, was positively associated with BMI and greater odds of obesity class I and obesity class II. Low wealth index was also associated with increased odds of underweight. Considering the large variance of the wealth index (our values ranged from one to 26), the magnitude of differences between predicted values of BMI is large. When the coefficient was applied to the values for the 10th and 90th percentile of wealth index values, the model predicted a difference of 4.57 kg for a typical man and 3.88 kg for a typical woman.

It is likely that SES acts is a distal factor and the exact mechanisms through which it may affect odds of underweight, overweight, and obesity are varied [67]. Families with higher socioeconomic status likely differ in their lifestyle—including dietary and physical activity patterns—thus affecting risk. While we examined these factors, the wealth index may have captured excess variability not completely accounted for by our assessments. Associations between socioeconomic markers (education, income, and possession-based wealth indices) and obesity have been found in other recent studies of rural individuals [6, 68]. Those of lower SES were more likely to be underweight, a result that is consistent with other studies in rural Tamil Nadu [69] and elsewhere in India [6]. This finding corresponds with the large body of research suggesting low socioeconomic status is associated with limited food intake and excessive manual labour, thus making it difficult for individuals to achieve net positive energy intake required to maintain or gain weight [6, 19, 34]. Such associations support the theory that social determinants play a role in both over- and undernutrition [70].

#### Livestock ownership

A unique finding of the present study was the inverse association between livestock ownership and BMI. Although livestock ownership was included in the wealth index, it was also independently associated with lower BMI when controlling for extemporaneous variables. Due to the lack of formal research on this topic, our knowledge of this association is limited to conjecture. In rural Tamil Nadu, most farmers graze their animals on forested public lands [71, 72], which often leads to individuals walking long distances every day to access fodder for their livestock. The result may be higher energy expenditure not fully captured by the PA score. Livestock ownership may also increase accessibility to milk products and lean meats such as chicken and mutton, thus resulting in increased consumption of animal proteins not fully captured in the FFQ. Regardless, this association was unexpected and merits further investigation on a larger scale.

#### Television ownership and TV time

Prevalence of television ownership was very high at 88.1 %. Although comparative data from other studies in India are rare, we found higher ownership rates than those reported by the 2006 National Readership Study, which found televisions in 76.2 % of households in Tamil Nadu [73]. Our findings are likely attributable to a populist scheme launched by the ruling Tamil Nadu state political party in 2006 to distribute colour televisions to the poor [74]. Over the following 4 years, the government purportedly delivered over 13.7 million televisions to Tamil Nadu residents [75].

Television ownership was associated with higher odds of overweight and class I obesity. This association is likely two-fold. First, television ownership may be indicative of overall wealth and SES. Although it was included in the wealth index, due to high prevalence of television ownership (Table 1) and its relatively low weight in the aggregate score, it may have had a small impact on wealth index and thus is also independently indicative of SES. This may indicate that some poor families were overlooked by the free TV scheme, or sold the televisions they received. Second, television ownership may encourage sedentary leisure time. Indeed, we found that time spent watching television (“TV time”) was associated with higher BMI and increased odds of class I obesity. TV time has been linked with overweight and obesity among children in other areas of India, however this is the first study to find such an association among adults [76]. Surprisingly, sitting time was not significantly associated with any outcome, contrary to findings in other countries [77]. It is possible that television ownership and TV time are more accurate indicators of sedentary leisure time than reported sitting time due to response bias.

#### High caste

High caste was associated with higher BMI and increased odds of obesity class I. These results correspond with those of Gaiha et al. (2010) [69], who found that low caste (including SC, ST, and other backwards caste (OBC)) was associated with lower risk of obesity. Similarly, Adinatesh et al. (2013) [78] found a higher prevalence of overweight and obesity among higher-caste individuals when compared to those identifying as SC, ST, or OBC. This association is likely complex, but we conjecture that it is likely due to two main categories of factors. The first is lifestyle factors. As seen by Adinatesh et al., high caste populations tend to have lower undernutrition, higher income and standards of living, greater access of sedentary pastimes (e.g. television, video games), and increased usage of vehicles for transportation. In addition, geneticists have recently proven the existence of genetic differences between castes, with higher castes having a higher proportion of West Eurasian genetic admixture [79]. Unfortunately, no research has yet examined caste genetics as they pertain to health and disease outcomes. Considering the roles of genetic factors in development of overweight and obesity [80], genetic differences between castes may play an important role in the association between caste and BMI. More research is needed to explore the ongoing role of caste and caste genetics in determining risk of overweight and obesity in rural areas.

#### Tobacco consumption

In India, tobacco is consumed in various forms. Cigarettes are growing in popularity, especially among urban and high-income populations [81]. Bidis (thin hand-rolled tobacco leafs) are cheaper and more commonly smoked among lower-income and rural populations. In addition, use of smokeless tobacco is pervasive among both men and women. Smokeless tobacco is often consumed as a combination of betel-leaf, areca nut, and tobacco called *paan* [82]. We found that any-type tobacco consumption was associated with lower BMI and lower odds of obesity class II. Tobacco use was also associated with increased odds of underweight. These findings are consistent with previous studies in the US [83–85] and urban India [86]. While the reasons behind this association are varied and complex, it is likely that tobacco may act directly (by affecting appetite and other aspects of physiology) or indirectly (by decreasing the purchasing power for food and therefore quality of diet) [82]. Considering the implications of tobacco consumption on both risk of underweight and risk of cancers and cardiovascular diseases, public health programs must emphasize preventing tobacco use and supporting cessation efforts among addicted individuals.

#### Dietary factors

India is experiencing a nutrition transition characterized by decreased consumption of coarse cereals and dietary diversification [44]. In some ways, this has benefited rural nutrition; data from the past 40 years suggest that fruit and vegetable consumption has doubled and overall protein intake has improved [4]. However, there have been significant drawbacks. Traditional small millets have been replaced by refined wheat and polished white rice due to imbalanced subsidization and shifting taste preferences [87]. Consumption of fats and sugars is also increasing due to the rising popularity of snack foods. We examined intake of foods, food groups, macro-nutrients, and micro-nutrients for potential associations with BMI, overweight, and obesity. We found significant associations with dietary intake of n-6 polyunsaturated fatty acids and animal fats.

### Oil consumption

The role of dietary fats in adult human obesity is a controversial issue [88]. A review by Willett (2001) [89] concludes that diets high in fat do not account for high prevalence of excess body fat in Western countries, and that the emphasis on total fat reduction has been a “serious distraction in efforts to control obesity and improve health in general” (pp. 59). His argument is based on both epidemiological evidence showing a poor association between total fat intake and obesity, and an assessment of published clinical trials that examine changes in fat composition in the diet. By contrast, a separate review conducted by Bray and Popkin (1998) [90] concludes that fat intake does promote obesity through passive overconsumption. Animal studies show a strong association between high-fat diets and obesity, and some studies on humans found that increasing fat intake was associated with an increase in BMI and higher odds of obesity [91, 92]. Bray and Popkin conclude that a reduction in dietary fat should be seen as a means to reduce to reduce total energy intake and reduce the energy density of the diet. The debate is inconclusive and as yet, there is no consensus in the literature about the association between fat intake and BMI, overweight, and obesity.

Evidence from the present study suggests that such reviews may disregard the qualitative composition of fat intake and the role of different types of fats. Intake of n-6 (also called omega-6) polyunsaturated fatty acids (PUFAs) was positively associated with BMI and negatively associated with odds of underweight. Ailhaud et al. (2006) claim that increased consumption of n-6 PUFAs, especially when combined with low intake of n-3 PUFAs, may promote development of excessive adipose tissue [86]. One clinical trial found that in elderly men fed a diet high in linoleic acid (LA, a n-6 PUFA), the mean body weight of the experimental group (*n* = 393) increased while the mean body weight of the control group (*n* = 389) decreased. Other studies have found that higher n-6 PUFA to n-3 PUFA intake ratios result in increased risk of several chronic diseases, including coronary heart disease, hypertension, type 2 diabetes, renal disease, rheumatoid arthritis, ulcerative colitis, Crohn’s disease, and chronic obstructive pulmonary disease, many of which have independent associations with adiposity [93]. Other beneficial effects of n-3 PUFAs include improvement of platelet aggregation, serum triglyceride concentrations, and anti-arrythmic effects. Some of these effects are directly related to reduced metabolic syndrome and obesity. Despite this, and despite independent connections between the metabolic syndrome and excess adiposity, the role of n-6 fatty acid intake in overweight and obesity is not confirmed nor well understood. Clearly, further studies are needed.

In India, n-6 PUFA intake has increased in recent years, especially in landlocked areas with little access to seafood [46]. While many studies recommend a n-6:n-3 ratio of at least 4:1 [93], the mean n-6:n-3 ratio in our study population was 24:1. Although n-6:n-3 intake ratio was not significantly associated with BMI or any outcome variables, our results indicate that n-6 intake may be associated with body weight in rural India. Considering the high intake of n-6 PUFAs in comparison to n-3 PUFAs and the wealth of data indicating the health benefits of n-3 PUFAs, food policies and health education programming aimed at increasing consumption of n-3 PUFAs would be beneficial. Such actions may also reduce prevalence of underweight by discouraging energy-dense and nutrition-poor diets in favor of those that are nutrition-dense and energy-adequate [94]

### Animal fat intake

We found animal fat intake (saturated fats in milk products and meat) was negatively associated with BMI. This trend conflicts with animal experiments showing that diets high in animal fats leads to obesity and insulin resistance [95]. Less research has been done in this area with humans. In adults, failing to control for factors such as physical activity or smoking often confounds studies. Some epidemiologic cross-sectional studies found associations between saturated fatty acid intake and BMI or other obesity outcomes [96–98]. Another cross-sectional epidemiology study found that obese children had higher intake of saturated fatty acids after controlling for physical activity and overall energy intake [99]. However, due to the cross-sectional nature and low sample size of these studies, it is difficult to reach any causative conclusions about the effects of animal fat intake on overweight and obesity. Therefore, although our results conflict with other studies, they also add to this debate and we encourage further research with larger sample sizes before making any broad conclusions about the effects of dietary fats on adiposity. We may suggest that certain authors, such as Gaiha et al. (2010) [69] are too hasty in their recommendations to limit saturated fats in favour of polyunsaturated fats.

This is the first cross-sectional epidemiological study, to our knowledge, to find significant linear associations between fatty acid intake and BMI while controlling for many extemporaneous risk factors. However, we found no significant association between n-6 PUFA and animal fat consumption and overweight, obesity class I, or obesity class II during the logistic regression analysis, while n-6 PUFA intake was associated with decreased odds of underweight. This may indicate that associations between fatty acid intake and BMI are driven by values within the underweight and normal range. In addition, average fat intake of the study population was well below values reported elsewhere in India [22] and also below recommendations for rural populations set out by the Indian National Institute of Nutrition [100], which may have skewed results. Further research is therefore needed in this field.

#### Limitations

Our study has a number of limitations. Most importantly, the cross-sectional nature of the study precludes the ability to distinguish causes from effects. BMI was used as a measure of adiposity instead of waist-to-hip ratio or triceps or subscapular skinfold thickness, which may be more valid methods of determining excess body fat and categorizing high-risk overweight and obesity [101]. However, BMI is more accurate when comparing individuals within the same ethnic group [101]. The choice of newer and more sensitive IOTF BMI cut-offs for determining overweight and obesity reduces the comparability of our data to previous studies in India. Several potential risk factors (e.g. family history of obesity, early childhood adiposity) were not assessed or controlled for since these data were not available. An FFQ was used for dietary assessments, and the validity of this approach has been documented by comparisons with more detailed methods [22]. However, FFQs are imperfect, are subject to recall bias, and do not reflect long-term dietary changes that may impact adiposity and disease risk [102].

## Conclusion

The epidemic of overweight and obesity is a serious public health issue in rural India and raises concerns about the capacity of the health care system to cope with associated outcomes. Alarming prevalence data, including those in the current study, provide a dire picture and a call to action. A population-level strategy to prevent obesity is necessary. Such a strategy requires sound data to reduce inefficiencies and target risk factors that have the greatest impact. While further research is needed, results from our study suggest several risk factors that are associated with BMI, overweight, and obesity, and may inform public health programming. Risk factors identified by our study may be divided into two categories. One category is individual-level risk factors that may be targeted by behaviour changing programming. Such factors included physical activity, television usage, and dietary factors such as finger millet, oil, and fat consumption. The second category comprises society-level risk factors such as caste and socioeconomic status. These risk factors must be addressed on a macro- level through social welfare policy, public infrastructure and development programs. As India continues to develop, modernize, and urbanize, there needs to be a strong education program to ensure that populations are aware of the dangers of overweight, obesity, and related noncommunicable diseases.

## Appendix 1

List of factors analyzed in all univariate regression analyses.

## Abbreviations

BMI: body mass index
CI: confidence interval
EU: European Union
FFQ: food frequency questionnaire
GPAQ: global physical questionnaire
MDRF: Madras Diabetes Research Foundation
OBC: other backwards castes
OR: odds ratio
PA: physical activity
PUFA: polyunsaturated fatty acid
TV: television
SC: scheduled caste
SES: socioeconomic status
ST: scheduled tribe
US: United States
WHO: World Health Organization
